# Rupture of a splenic artery aneurysm in a previously healthy 53-year-old male

**Published:** 2014

**Authors:** A Papadomichelakis, D Anyfantakis, M Kastanakis, P Karona, E Bobolakis

**Affiliations:** * First Surgery Department, Saint George General Hospital, Chania, Crete, Greece; ** Primary Health Care Centre of Kissamos, Chania, Crete, Greece

**Keywords:** Splenic artery aneurysm, rupture, diagnosis

## Abstract

Splenic artery aneurysms are unusual clinical conditions that may be ruptured resulting into adverse health outcomes. Pregnancy, portal hypertension and atherosclerosis are conditions that predispose to the formation of splenic artery aneurysms. A rare case of a previously healthy man referred to our department by his general practitioner complaining of acute abdominal pain is presented. During the hospital stay, the patient presented hemodynamic instability. Abdominal computed tomography disclosed perihepatic and perisplenic fluid accumulation. A diagnosis of ruptured splenic artery aneurysm was performed and the patient was operated successfully with splenectomy and ligation of the splenic artery. Although the condition is rare, physicians have to be aware of the high mortality rates caused by a ruptured splenic artery aneurysm and include this in the differential diagnosis when they encounter patients with acute abdominal pain and hemodynamic instability.

## Introduction

Visceral artery aneurysms represent relatively unusual clinical entities [**1**]. The aneurysm of the splenic artery is the third most common site among intra-abdominal [**2**] and the most frequent among visceral artery aneurysms (60%) [**1**]. Their rupture is associated with a high mortality related burden [**3**]. A case of ruptured splenic artery aneurysm in a previously healthy 54-year-old male is presented.

## Case presentation

A 53-year-old Caucasian Greek male was referred by his general practitioner to the emergency department of the Saint George General Hospital of Chania, Crete because of a 3-hour history of acute abdominal pain located in the epigastric region, without radiation, accompanied by nausea. His past medical history was unremarkable and negative for abdominal trauma, pancreatitis, chololitiasis or previous surgery. The patient was hemodynamically stable. His vital signs on admission were the following: temperature, 36.8 degrees Celsius; blood pressure, 140/90 mmHg; heart rate, 75 beats/min; oxygen saturation, 98%, while he was breathing ambient air; temperature, 36.5. Abdominal examination disclosed tenderness upon the left upper quadrant. 

**Fig. 1,2:**
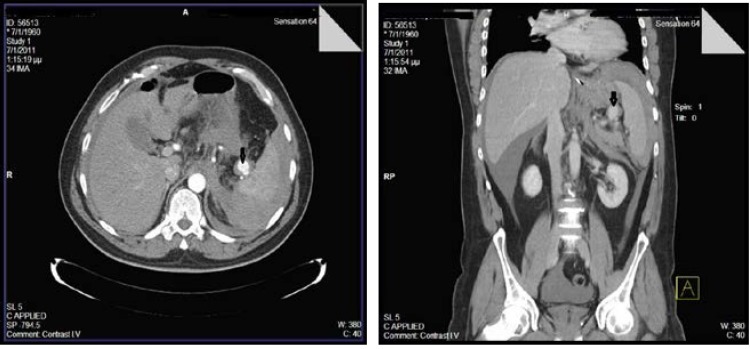
Perihepatic and perisplenic hemorrhage secondary to a ruptured splenic artery aneurysm (black arrows)

The initial laboratory work up showed: white blood count, 10.72 cells/μl (normal range: 4-11 cells/μl); hematocrit, 35% (normal range: 40-50%); haemoglobin 11.4 g/dl (normal range: 13.5-17.5 g/dl); platelet counts, 267 cells/μl (normal range: 150-450 cells/μl). Abdominal ultrasonography was normal except for a moderate enlargement of the spleen (diameter: 13.5 cm). Eight hours after the admission, the patient presented pallor, agitation, signs of hypovolemic shock with tahypnoea (22 breaths/min), hypotension (80/50 mmHg) and tachycardia (120 beats/minute). The complete blood count disclosed hemodynamic instability with a considerable drop of hematocrit (27.8%) and of hemoglobin (9g/dl). Computed tomography of the abdomen with intravenous contrast enhancement was consecutively performed showing the presence of hemoperitoneum due to the rupture of a splenic artery aneurysm (**Fig. 1,2**).

Urgent laparotomy with splenectomy and ligation of the splenic artery proximal to the aneurysm controlled the bleeding diathesis. Three units of red blood cells were administered. The patient was transferred to the intensive care unit for 24 hours. He had an uneventful postoperative recovery and was discharged home in the 10th hospital day.

## Discussion

Splenic artery aneurysms are unusual clinical entities [**1**]. Incidence rates range from 0.02% to 0.1% with a predilection in 5th and 6th decades of life and female gender [**2**]. They may be either congenital or secondary to conditions such as hypertension, portal hypertension, cirrhosis [**4**], liver transplantation, pregnancy [**5**] and chronic pancreatitis [**6**]. Our patient did not present any of the previously mentioned risk factors. 

The majority of patients with splenic artery aneurysms are asymptomatic [**5**] and the diagnosis is usually performed incidentally during abdominal ultrasound imaging or post-mortem examination [**1**]. The rupture of the splenic aneurysm is the most severe complication and usually occurs in the final trimester of pregnancy [**1**]. Remarkably, mortality rates after rupture range between 25-70% [**3**]. Epigastric pain is the most prevalent clinical manifestation [**1**]. Rupture is accompanied by free intra-peritoneal hemorrhage, hypovolemic shock and collapse [**1**].

The surgical management of a ruptured splenic artery aneurysm consists of aneurysm resection, with or without splenectomy [**7**]. The use of splenic angioembolization has also been reported as a safe and effective alternative in hemodynamically stable patients [**8**].

The diagnosis of splenic artery aneurysm was difficult to suspect in our patient. Although the rupture of a splenic artery aneurysm is rare, physicians must always cultivate a “detective judgment” [**9**] and include this fatal condition in the differential diagnosis of acute abdominal pain and hypovolemic shock. 

**Consent**

A written informed consent was obtained from the patient for the publication of this case report and accompanying images. A copy of the written consent is available for review by the Editor-in-Chief of this journal.

**Conflict of Interests**

The authors declare that they have no competing interests.
